# Esaxerenone Attenuates Aldosterone-Induced Mitochondrial Damage-Mediated Pyroptosis in Mouse Aorta and Rat Vascular Smooth Muscle Cells

**DOI:** 10.3390/life14080967

**Published:** 2024-07-31

**Authors:** Yunqian Xian, Xuan Wang, Yi Chang, Panpan Qiang, Yutong Han, Juan Hao, Xiaomeng Gao, Tatsuo Shimosawa, Qingyou Xu, Fan Yang

**Affiliations:** 1 Graduate School, Hebei University of Chinese Medicine, Shijiazhuang 050200, China; xianyunqian@hebcm.edu.cn (Y.X.); wangxuan@hebcm.edu.cn (X.W.); qiangpanpan@hebcm.edu.cn (P.Q.); hanyutongzy@163.com (Y.H.); 15732190326@163.com (X.G.); 2 Hebei Key Laboratory of Integrative Medicine on Liver-Kidney Patterns, Hebei University of Chinese Medicine, Shijiazhuang 050200, China; changyiyi@hebcm.edu.cn (Y.C.); haojuan2903@163.com (J.H.); 3 Institute of Integrative Medicine, College of Integrative Medicine, Hebei University of Chinese Medicine, Shijiazhuang 050200, China; 4 Department of Clinical Laboratory, School of Medicine, International University of Health and Welfare, Narita 286-8686, Chiba, Japan; tshimo-tky@umin.ac.jp

**Keywords:** aldosterone, esaxerenone, mitochondria, pyroptosis, vascular smooth muscle cell

## Abstract

Background: Vascular smooth muscle cell (VSMC) injury caused by the inflammatory response plays a key role in cardiovascular disease (CVD), and the vasoprotective effects of mineralocorticoid receptor blockers (MRBs) support the role of mineralocorticoid receptor (MR) activation. Methods: C57BL/6 mice and VSMCs isolated from rats were treated with aldosterone and esaxerenone. Caspase-1, GSDMD-N, IL-1β, and NR3C2 expression and DNA damage in aortic VSMCs were detected using immunohistochemistry, Western blotting, and TUNEL staining. Mitochondrial changes were detected by transmission electron microscopy (TEM). Reactive oxygen species (ROS), MitoTracker, JC-I, mitochondrial respiratory chain complexes I–V, and NR3C2 were detected using immunofluorescence and flow cytometry. Pyroptosis was detected with scanning electron microscopy (SEM). Results: After aldosterone treatment, the number of TUNEL-positive cells increased significantly, and the expression of caspase-1, GSDMD-N, and IL-1β increased. TEM revealed mitochondrial damage, and SEM revealed specific pyroptotic changes, such as cell membrane pore changes and cytoplasmic extravasation. Increased ROS levels and nuclear translocation of NR3C2 were also observed. These pyroptosis-related changes were reversed by esaxerenone. Conclusions: Aldosterone activates the MR and mediates mitochondrial damage, thereby inducing pyroptosis in VSMCs via the NLRP3/caspase-1 pathway. Esaxerenone inhibits MR activation and reduces mitochondrial damage and oxidative stress, thereby inhibiting pyroptosis.

## 1. Introduction

The number of prevalent cases of total cardiovascular disease (CVD) nearly doubled from 271 million in 1990 to 523 million in 2019, and CVD has become the main disease burden worldwide [1]. Hypertension represents a significant risk factor for CVD and remains the primary cause of premature mortality on a global scale; thus, the prevention and control of hypertension is an important global public health strategy in efforts to reduce premature mortality from CVD. As the vasodilatation capacity plays a crucial role in regulating blood pressure, vascular damage is the cause of most types of CVD [2]. Cell death and sterile inflammation, which include pyroptosis, a type of programmed cell necrosis accompanied by an inflammatory response, are now thought to play important roles in the development of CVD [3]. Previous studies have shown that pyroptosis is involved in various CVDs, such as hypertension and atherosclerosis [4,5], and involves macrophages and endothelial cells [6,7]. Recent studies have indicated that vascular injury occurs not only because of changes in the intima of blood vessels but also because vascular smooth muscle cells (VSMCs) play important roles in vascular inflammation and CVD progression [4,8]. Although an increasing number of studies have focused on the role of VSMCs in CVD, the detailed molecular mechanisms underlying cardiovascular injury caused by VSMC pyroptosis remain elusive.

Many factors contribute to VSMC damage, including elevated calcium levels [9,10], phosphate levels [11], and oxidative stress [12]. Recent studies support the role of mitochondrial adaptors, mitochondrial calcium fluxes, and mitochondrial reactive oxygen species (mtROS) generation in inflammasome activation, in which mitochondrial-derived mtROS are the primary source of cellular ROS, and excessive mtROS can mediate pyroptosis and promote atherosclerosis by activating the NOD-like receptor thermal protein domain-associated protein 3 (NLRP3) inflammasome and modulating its downstream molecules caspase-1 and interleukin-1β (IL-1β) [8]. Accumulating evidence suggests that aldosterone, in addition to regulating blood pressure and electrolyte homeostasis, has direct vascular effects that promote cardiovascular remodeling in individuals with hypertension [13]. Aldosterone and oxidative stress may be important regulators of vascular changes associated with hypertension [14]. However, whether aldosterone can cause aortic injury through VSMC pyroptosis requires further verification.

Numerous studies have established that aldosterone regulates cardiovascular system homeostasis by interacting with the mineralocorticoid receptor (MR) to activate specific intracellular genomic and nongenomic pathways [15]. Thus, the blockade of MRs is clinically effective at preventing hypertension, cardiovascular and renal disease [16]. Esaxerenone is a novel nonsteroidal and selective MR blocker for the treatment of hypertension [17]. Previous studies have shown that esaxerenone inhibits aldosterone binding to MRs with greater potency than spironolactone and eplerenone [18]. However, the potential therapeutic value of esaxerenone in CVD is not yet clear, and further exploration is needed.

We hypothesized that aldosterone promotes mitochondrial oxidative damage and pyroptosis in VSMCs, which may contribute to aortic injury and hypertension. In this study, we investigated the effects of aldosterone and esaxerenone on VSMC pyroptosis, providing insights into the prevention of mitochondrial damage-induced cell death and the progression of hypertension.

## 2. Materials and Methods

### 2.1. Animals and Experimental Design

Thirty male SPF C57BL/6 mice aged 6 weeks and weighing 20 ± 2 g (Liaoning Changsheng Biotechnology Co., Ltd., Liaoning, China, SCXK (Liao) 2020–0001) were used in this study. The mice were maintained on standard chow and tap water at 25 °C under a 12-h light/dark cycle. All experiments were performed in accordance with the recommendations for the Care and Use of Laboratory Animals in the National Institutes of Health Guidelines. All animal care and experimental protocols met the standards of the Animal Experiment Ethics Committee of Hebei University of Chinese Medicine (Hebei, China).

After 7 days of adaptive feeding, the mice were randomly divided into 3 groups (*n* = 10 mice/group): the CON group, the ALD group, and the ESA group. The CON group was perfused with the vehicle (0.5% ethanol). For the ALD group, aldosterone was dissolved in absolute ethanol at 0.1 mg/mL, a mini-osmotic pump (ALZET model 2006, DURECT Corporation, Cupertino, CA, USA) was subcutaneously embedded, and aldosterone (CAS NO. 52-39-1, Cayman Chemical, Ann Arbor, MI, USA) was continuously perfused at 0.75 μg/h according to previous research and the manufacturer’s instructions. For the ESA group, aldosterone-infused mice were orally treated with esaxerenone (kindly provided by Daiichi Sankyo Co., Ltd., Tokyo, Japan), at a dose of 1 mg/kg/d according to the relevant literature and manufacturer’s instructions [19,20]. After 12 weeks, the mice were anesthetized with a mixture of 3% isoflurane and oxygen, and the aortas were collected for histological analysis.

### 2.2. Cell Culture

Rat VSMCs were purchased from the BeNa Culture Collection (Xinyang, Henan Province, China). The cells were grown in 90% Dulbecco’s modified Eagle’s medium (DMEM)-H + 10% fetal bovine serum (FBS) (SA211.02, CellMax, China) in an incubator at 37 °C with 5% CO_2_. When the cell confluence reached 50%–70%, the cells were randomly divided into 3 groups: the CON group, the ALD group, and the ESA group. After cell adherence, the culture medium was replaced with serum-free medium, and the cells were incubated for 24 h to render them quiescent. Normal medium was added to the CON group. Aldosterone (10^−7^ mol/L) was administered to the ALD group according to the previous research and the manufacturer’s instructions [19]. For the ESA group, esaxerenone was dissolved in dimethyl sulfoxide (DMSO) at a concentration of 10^−3^ mol/L and then added to the culture medium at a final concentration of 10^−6^ mol/L 2 h before the addition of aldosterone, and then, the cells were treated with a final concentration of 10^−7^ mol/L aldosterone as recommended by the manufacturer [19]. The cells were collected after 24 h for subsequent experiments.

### 2.3. Histopathological Analysis and Immunohistochemistry Analysis

Aortas were fixed with 4% paraformaldehyde (PFA) overnight, dehydrated with alcohol, and embedded in paraffin. The paraffin blocks were cut into 5 μm sections for hematoxylin and eosin (H&E) staining, Masson’s trichrome staining, and immunohistochemical analysis of nuclear receptor subfamily 3 group C member 2 (NR3C2) (1:100, Proteintech, China, Cat#: 21854-1-AP), NLRP3 (1:50, Novus, USA, Cat#: NBP2-12446), caspase-1 (1:100, Proteintech, China, Cat#: 22915-1-AP), gasdermin D (GSDMD)-N (1:100, Proteintech, China, Cat#: AF4012), and IL-1β (1:100, Abcam, UK, Cat#: ab9722) expression. A light microscope (BX53, Olympus, Japan) was used for observation and image capture. The grayscale values were quantified using ImageJ 1.53e software (National Institutes of Health, Bethesda, MD, USA).

### 2.4. TdT-Mediated dUTP Nick End Labeling (TUNEL) Staining

Paraffin-embedded mouse aortic sections were examined using an In Situ Cell Death Detection Kit, POD (45197300, Roche, Basel, Switzerland), according to the manufacturer’s instructions. After the aortic tissue was fixed, embedded, sectioned, and deparaffinized, a proteinase K solution (20 μg/mL, P1120, Solarbio, China) was added dropwise. After incubation at 37 °C for 30 min, 50 μL of terminal-deoxynucleotidyl transferase-mediated dUTP nick end labeling (TUNEL) reaction mixture (prepared with the enzyme solution and labeling solution at a ratio of 1:9) was added, and the mixture was incubated at 37 °C for 60 min. After adding a peroxidase converter and incubating at 37 °C for 30 min, the nuclei were developed with DAB and counterstained with hematoxylin. A light microscope was used for observation.

For the cell experiments, the cell samples in 24-well plates were fixed, blocked, and permeabilized after collection. Then, 150 μL of TUNEL reaction mixture (prepared with 1:9 enzyme solution and labeling solution) was added, and the mixture was incubated at 37 °C for 60 min. Finally, the slides were observed and analyzed under a fluorescence microscope (EVOS FL Auto, Thermo Fisher, USA). TUNEL-positive cells were counted, and the percentage of TUNEL-positive cells was quantified via histogram analysis, with 6 images analyzed per group.

### 2.5. DNA Damage Detection (γ-H2AX Immunofluorescence Staining)

For the cell-based experiments, the degree of DNA damage was measured with a DNA damage detection kit (C2035S, Beyotime, Nantong, China). The cell samples were washed with PBS, the fixative solution was added, and the cells were fixed for 15 min. The fixative solution was aspirated, and the cells were washed 3 times with the washing solution for 5 min each. Immunostaining blocking solution was added, and the cells were blocked for 20 min at room temperature. The immunostaining blocking solution was aspirated, the γ-H2AX rabbit monoclonal antibody was added, and the cells were incubated for 1 h at room temperature. The cells were subsequently washed 3 times with the washing solution for 10 min each. An anti-rabbit 488 antibody was added, and the mixture was incubated for 1 h at room temperature. The cells were subsequently washed 2 times with washing solution for 10 min each. Nuclear staining solution (DAPI) was added, and the cells were incubated at room temperature for approximately 5 min. The nuclear staining solution was aspirated, and the cells were washed 3 times with the washing solution for 5 min each. The slides were sealed with coverslips and observed under a fluorescence microscope.

### 2.6. Transmission Electron Microscopy (TEM)

For tissue samples, fresh mouse aortas were removed and quickly placed in electron microscopy fixative solution (G1102, Servicebio, China) at 4 °C for 3 h. The samples were then fixed with 1% osmic acid·0.1 mol/L phosphate buffer (PB) at 20 °C for 2 h. The tissues were dehydrated in a graded series of alcohol solutions and treated for 3 h in acetone and 812 embedding medium (90529-77-4, Structure Probe, Inc., West Chester, PA, USA) prepared at a ratio of 1:1. The samples were then placed in acetone and 812 embedding media prepared at a ratio of 1:2 and infiltrated overnight. Pure 812 embedding medium was added for 7 h. Pure 812 embedding medium was poured into an embedding plate, the samples were placed into the embedding plate, and the plate was placed in an oven at 37 °C overnight. The samples then underwent polymerization in a 60 °C oven for 48 h. The tissues were cut into 60-nm ultrathin sections using an ultramicrotome (Leica UC7, Leica, Germany). After double staining with uranium and lead (2% uranyl acetate saturated alcohol solution and lead citrate, with 15 min of staining with each solution), the sections were dried at room temperature overnight. Observations and image analysis were performed via transmission electron microscopy (TEM) (HT7700, HITACHI, Japan).

For the cell samples, the culture medium was discarded, and the cells were incubated with electron microscopy fixative for 2 h at 4 °C. The cells were subsequently centrifuged at low speed, embedded in 1% agarose, and rinsed 3 times with 0.1 mol/L PB for 15 min each time. The subsequent processing steps were the same as those for the tissue samples. TEM was then used for observation and image analysis.

### 2.7. Scanning Electron Microscopy (SEM)

PBS (G0002, Servicebio, China) was used to rinse the cells, the PBS was discarded, and the cells were fixed with electron microscopy fixative solution (G1102, Servicebio, China) at room temperature for 2 h and then stored at 4 °C. The fixed samples were rinsed 3 times with 0.1 mol/L phosphate buffer (PB, pH 7.4) for 15 min each. The cells were then fixed with 1% osmic acid in 0.1 mol/L phosphate buffer (PB) at 20 °C for 1–2 h. Next, 0.1 mol/L phosphate buffer (PB, pH 7.4) was added, and the samples were rinsed 3 times for 15 min each. The cells were incubated with 30%, 50%, 70%, 80%, 95%, and 100% alcohol for 15 min each time and then with isoamyl acetate (Sinopharm Chemical Reagent Co., Ltd., China) for 15 min. The cells were placed inside a critical point dryer (K850, Quorum, USA) for drying and then treated electrically with a sputter coater (MC1000, HITACHI, Japan). The cells were then observed with a scanning electron microscope (SU8100, HITACHI, Japan).

### 2.8. Flow Cytometry

VSMC apoptosis was examined with a PE Annexin V Apoptosis Detection Kit I (559763, BD Pharmingen, USA). After the cells were collected and washed with cold phosphate-buffered saline (PBS), they were resuspended in 1× binding buffer (diluted according to the manufacturer’s instructions) at a concentration of 1 × 10^6^ cells/mL. Then, 100 μL of the solution (1 × 10^5^ cells) was transferred to a 5 mL culture tube, and 5 μL of phycoerythrin (PE)-conjugated Annexin V (component no. 51-65875X) and 5 μL of 7-amino-actinomycin (7-AAD, component no. 51-68981E) were added. The cells were mixed gently and incubated in the dark at 25 °C for 15 min. The cells were then analyzed with a flow cytometer (BD FACSAria^TM^ Fusion, BD Biosciences, USA), and the data were further analyzed with BD FACSDiva^TM^ software (BD FACSDiva^TM^ software v7.0, BD Biosciences, Franklin Lakes, NJ, USA).

The reactive oxygen species (ROS) content of the VSMCs was detected with a dichlorodihydrofluorescein diacetate (DCFH-DA) assay (85155, Cayman Chemical, USA). The cells in the ALD group were stimulated with aldosterone for 20 min. The cells in the ESA group were pretreated with esaxerenone for 2 h before aldosterone stimulation. After the cells were collected, they were suspended in diluted DCFH-DA (at a final concentration of 10^−5^ mol/L) at concentrations ranging from 1 million to 20 million cells/mL and mixed well to ensure full contact between the probe and the cells. The cells were then incubated at 37 °C for 20 min in the dark. After the cells were washed, they were detected with a flow cytometer (BD FACSCalibur^TM^, BD Biosciences, USA). The detected data were analyzed with FlowJo 10 (BD FlowJo^TM^, Becton, Dickinson & Company, USA).

### 2.9. Immunofluorescence Analysis

For the immunofluorescence analysis, the cells were split into 24-well plates at a density of 1 × 10^5^ cells/mL, and drug interventions were administered as described above. The slides were fixed with precooled PFA for 10 min. After fixation, the cells were permeabilized with 0.5% Triton X-100. The cells were then washed with PBS and treated with 10% normal goat serum for 1 h at room temperature to reduce nonspecific antigen–antibody interactions. Primary antibodies against NR3C2 (1:200) and GSDMD-N were added, and the samples were incubated for 24 h at 4 °C. The secondary antibody (goat anti-rabbit IgG H&L (Alexa Fluor^®^ 488/555)), which was conjugated to Fluor 488 (GSDMD-N)/555 (NR3C2) (1:300, Abcam, Cambridge Science Park in Cambridge, UK), was added and incubated for 1 h at 37 °C. After the cells were washed with PBS, the samples were incubated with 4′6-diamino-2-phenylindole (DAPI, C0065, Solarbio, China) for 10 min at room temperature to stain the nuclei. A confocal microscope (Leica SP8, Leica, Germany) was used to observe the cells and capture images.

### 2.10. Observation of Cellular Reactive Oxygen Species (ROS) Production

Intracellular ROS were detected with a reactive oxygen species assay kit (S0033, Beyotime, Nantong, China). VSMCs were seeded into 24-well plates (1 × 10^4^ cells/well) in DMEM and grown to 70% confluence. After serum starvation overnight, the cells were washed with fresh serum-free DMEM and incubated with 10^−5^ mol/L DCFH-DA for 20 min (the optimal drug incubation time was selected according to the results of a preliminary experiment) at 37 °C. After the cells were washed 3 times with serum-free medium, the ALD group was incubated with 10^−7^ mol/L aldosterone for 20 min, and the ESA group was pretreated with 10^−6^ mol/L esaxerenone for 2 h. After the medium was changed, the cells were observed with a fluorescence microscope (EVOS FL Auto, Thermo Fisher, USA), and the fluorescence intensity was further analyzed using ImageJ 1.53e software, with 6 images analyzed per group.

### 2.11. Labeling with MitoTracker Red CMXRos

MitoTracker Red CMXRos was used to observe changes in the mitochondrial morphology. After VSMCs were treated with or without aldosterone and esaxerenone, they were incubated with MitoTracker Red CMXRos (C1049B, Beyotime, Nantong, China) for 30 min at 37 °C. The cells were washed with serum-free medium, and the medium was replaced with normal medium. Confocal microscopy (CSIM 110, SUNNY, China) was used to observe cells and photograph the cells.

### 2.12. Mitochondrial Membrane Potential Measurement

JC-I is widely used to detect the mitochondrial membrane potential as a dye that is located only in the mitochondria of living cells. In the presence of a normal mitochondrial membrane potential, J-aggregates form and produce red fluorescence. A lack of the mitochondrial membrane potential leads to the reduced formation of J-aggregates, resulting in green fluorescence [21].

VSMCs were incubated with the mitochondrial fluorescent probe JC-I (1×) (C2003S, Beyotime, Nantong, China) for 20 min at 37 °C after being treated with aldosterone or esaxerenone, according to the assigned treatment group. The cells were washed with Hanks’ balanced salt solution (with Ca^2+^ and Mg^2+^), and the medium was replaced with normal medium. The fluorescence intensity was observed using a confocal microscope and statistically analyzed using ImageJ, with 6 images analyzed per group.

### 2.13. Protein Extraction and Western Blotting

The cells and aortas were lysed in cold radioimmunoprecipitation assay (RIPA) buffer (BB-3201, Bestbio, China). Then, 20–30 μg of protein from whole-cell lysates or nuclear extracts was denatured in boiling water for 10 min. The samples were separated by sodium dodecyl sulfate–polyacrylamide gel electrophoresis (SDS–PAGE) and transferred to polyvinylidene fluoride (PVDF) membranes (R1NB77899, Merck Millipore Ltd., Tullagreen, Carrigtwohill, Co., Cork, Ireland). After blocking with 5% nonfat milk, the membranes were incubated overnight at 4 °C with primary antibodies against NR3C2 (1:1000, Proteintech, China, Cat#: 21854-1-AP and Abcam, UK, Cat#: ab64457 for cells and aortas, respectively), NLRP3 (1:300, Novus, USA, Cat#: NBP2-12446), caspase-1 (1:1000, Proteintech, China, Cat#: 22915-1-AP), GSDMD (1:1000, Affinity, China, Cat#: AF4012), IL-1β (1:1000, Affinity, China, Cat#: AF4006 and Abcam, UK, Cat#: ab9722 for cells and aortas, respectively), glyceraldehyde-3-phosphate dehydrogenase (GAPDH) (1:1000, Proteintech, China, Cat#: 60004-1-Ig), β-actin (1:1000, Proteintech, China, Cat#: 66009-1-Ig), β-Tubulin (1:1000, Affinity, China, Cat#: DF7967), PCNA (1:5000, Proteintech, China, Cat#: 60097-1-Ig) or histone-H3 (1:1000, Proteintech, China, Cat#: 17168-1-AP). The next day, the membrane was incubated with a secondary antibody (1:15,000, IRDye 680RD/IRDye 800CW, LI-COR Biosciences, USA) for 1 h at 25 °C. The signal was detected with an Odyssey Infrared Imaging System (LI-COR Systems, Lincoln, NE, USA) and quantified with ImageJ 1.53e software.

### 2.14. Mitochondrial Complex Activity Assay

For mitochondrial respiratory chain complexes I–V (COX I–V) activity assays in VSMCs, cells were harvested and assayed using a Mitochondrial Complex Activity Detection Kit (BC0515, BC3235, BC3245, BC0945, and BC1445, Solarbio, China) with a microplate reader (Molecular Devices, Silicon Valley, CA, USA) according to the manufacturer’s instructions. The protein concentration of each sample was detected with a BCA protein concentration assay kit (SW101-02, SEVEN BIOTECH, China). The activity of the complex was calculated via spectrophotometric analysis of the samples in microquartz cuvettes and was analyzed statistically.

### 2.15. Statistical Analysis

The results were analyzed with IBM SPSS 26.0 statistical software (IBM, Armonk, NY, USA). All the data are presented as the means ± standard deviations (SDs). Statistical comparisons between groups were performed using the Student’s *t*-test for two groups or one-way analysis of variance (ANOVA) followed by Tukey’s post hoc tests for multiple groups, and a *p*-value < 0.05 was considered to indicate statistical significance.

## 3. Results

### 3.1. Aldosterone-Induced Aortic Injury

**Figure 1:**
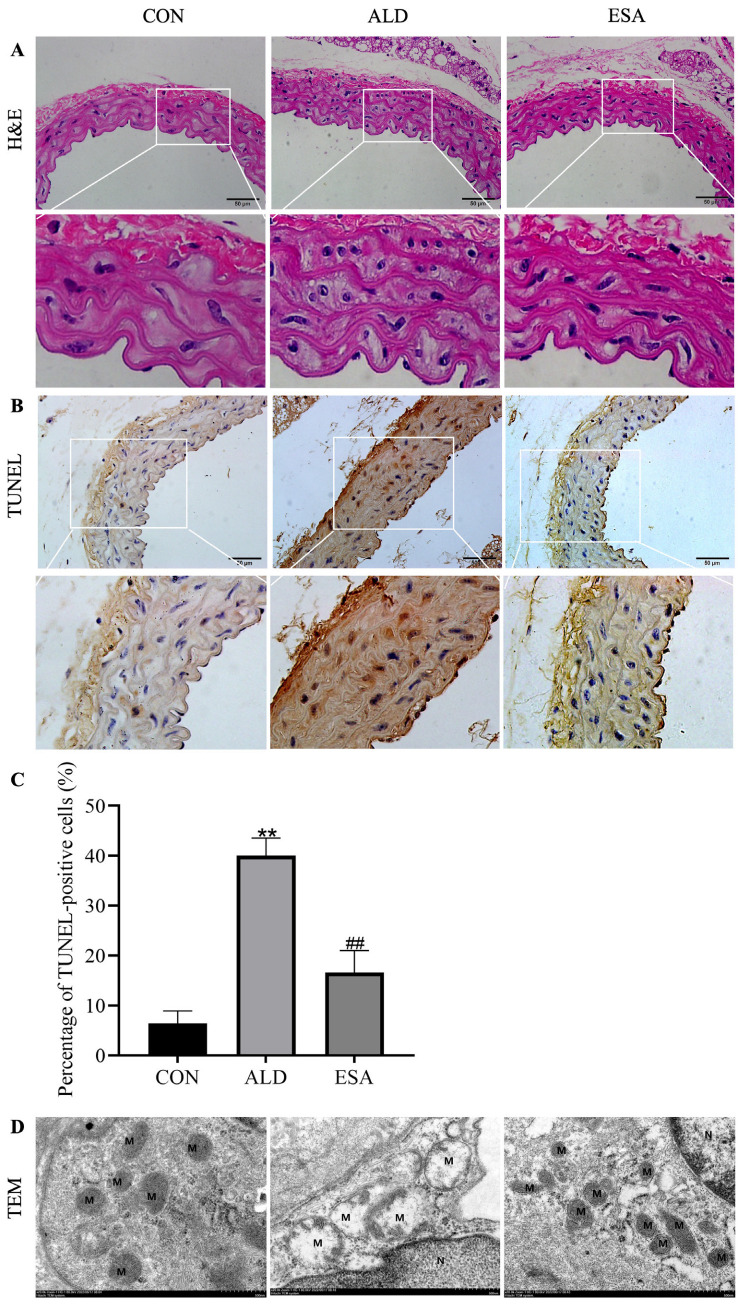
Effects of aldosterone on aortic injury. (**A**) Representative image of H&E staining used to assess aldosterone-induced pathological damage to aortic vascular smooth muscle cells (VSMCs). Scale bar = 50 μm. (**B**,**C**) TUNEL staining was used to assess DNA damage in aldosterone-perfused mouse aortic VSMCs, and the graphs show the percentage of TUNEL-positive cells. Scale bar = 50 μm. (**D**) Mitochondrial damage in aldosterone-perfused mouse aortic VSMCs was detected using transmission electron microscopy (TEM). M: Mitochondria; N: nucleus. Scale bar = 500 nm. *n* = 6. The values are the means ± SDs, ** *p* < 0.01 compared to CON, and ^##^ *p* < 0.01 compared to ALD. ALD: aldosterone perfusion. After 7 days of adaptive feeding, an osmotic minipump (ALZET model 2006, DURECT Corporation, Cupertino, CA, USA) was implanted (s.c.) to infuse aldosterone (0.75 μg/h) for 12 weeks. ESA: aldosterone-perfused mice were administered esaxerenone (kindly provided by Daiichi Sankyo Co., Ltd., Tokyo, Japan) at 1 mg/kg/d.

We performed H&E staining on aortic tissues to determine the effect of aldosterone on the aorta. Compared to those in the aortas of the mice in the CON group, the nuclei of the VSMCs in the ALD group were pyknotic, and after treatment with the aldosterone receptor blocker esaxerenone, aldosterone-induced aortic tissue damage was alleviated (Figure 1A). TUNEL staining revealed more severe DNA damage in aldosterone-infused mouse aortic VSMCs than in aortic VSMCs from the CON and ESA groups. The percentage of TUNEL-positive cells relative to the total number of cells was 6.448% ± 2.479% in the CON group, 40.0% ± 3.564% in the ALD group, and 16.592% ± 4.431% in the ESA group (Figure 1B,C). Changes in the mitochondrial ultrastructure were observed via TEM to assess whether the damage was related to mitochondrial damage. In terms of the ultrastructure of the mitochondria, the mitochondria of the VSMCs were severely swollen after stimulation with aldosterone. The intramembrane matrix was dissolved and ultimately disappeared, and the cristae were broken, missing, and/or vacuolized. However, esaxerenone effectively alleviated mitochondrial damage in the aortic VSMCs of aldosterone-infused mice (Figure 1D). We monitored the blood pressure of each group of mice postoperatively but did not observe significant changes [22]. Therefore, we hypothesized that aortic and VSMC damage occurred in aldosterone-perfused mice and that this damage was related to the mitochondrial pathway. Treatment with esaxerenone can attenuate aldosterone-induced aortic injury in mice.

### 3.2. Aldosterone-Induced Changes in Pyroptosis-Related Indicators in Aortic VSMCs

**Figure 2:**
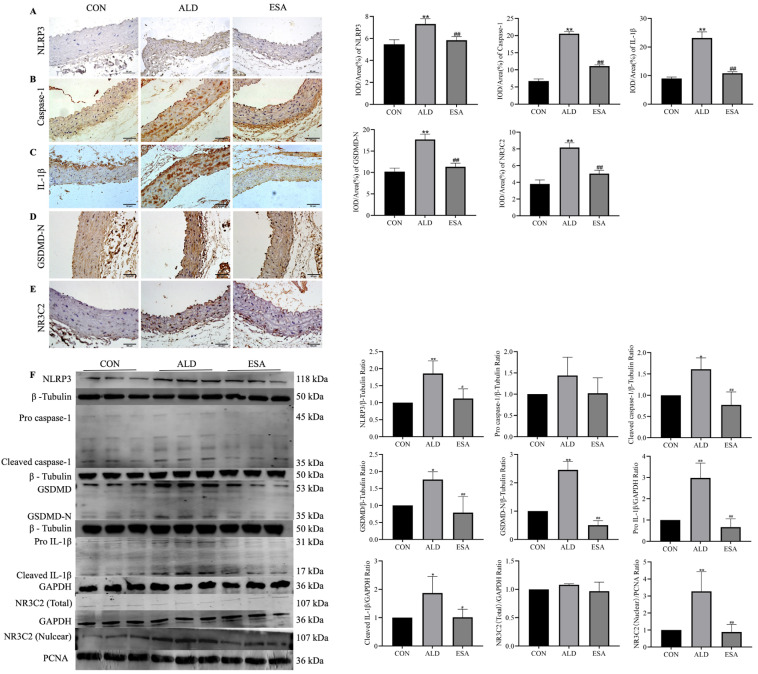
Effects of aldosterone on pyroptosis-related indicators in the aortic vasculature. Immunohistochemical staining for (**A**) NLRP3, (**B**) caspase-1, (**C**) gasdermin D (GSDMD)-N, (**D**) IL-1β, and (**E**) NR3C2 in aldosterone-perfused mouse aortic VSMCs to examine pyroptosis. (**F**) NLRP3, caspase-1, GSDMD-N, IL-1β and NR3C2 protein expression was detected via Western blotting. Scale bar = 50 μm. *n* = 6. The values are the means ± SDs, * *p* < 0.05 compared to CON, ** *p* < 0.01 compared to CON, ^#^ *p* < 0.05 compared to ALD, and ^##^ *p* < 0.01 compared to ALD. ALD: aldosterone perfusion. After 7 days of adaptive feeding, an osmotic minipump (ALZET model 2006, DURECT Corporation, Cupertino, CA, USA) was implanted (s.c.) to infuse aldosterone (0.75 μg/h) for 12 weeks. ESA: aldosterone-perfused mice were administered esaxerenone (kindly provided by Daiichi Sankyo Co., Ltd., Tokyo, Japan) at 1 mg/kg/d.

Pyroptosis-related indices were detected via immunohistochemical analysis to determine whether pyroptosis occurred in VSMCs in the aorta. The results revealed that the expression of NLRP3, caspase-1, IL-1β, GSDMD-N, and NR3C2 was greater in the ALD group than in the CON group. NLRP3 was expressed mainly in the cytoplasm and nucleus (Figure 2A), and caspase-1 and IL-1β were expressed mainly in the cytoplasm (Figure 2B,C). The pyroptosis-mediating gene GSDMD-N also exhibited the same trend in expression in each group (Figure 2D). The expression of the aldosterone receptor MR (NR3C2) was significantly increased, and NR3C2 translocated to the nucleus (Figure 2E). The expression of these indicators was lower in the ESA group than in the ALD group. The results of Western blotting for NLRP3, pro caspase-1, cleaved caspase-1, GSDMD, GSDMD-N, pro IL-1β, cleaved IL-1β, and NR3C2 (nuclear) showed the same trend as the results described above (Figure 2F). These results reveal the role of aldosterone-induced pyroptosis in VSMCs and the role of MR activation in this process.

### 3.3. Aldosterone Induces Pyroptosis in VSMCs

**Figure 3:**
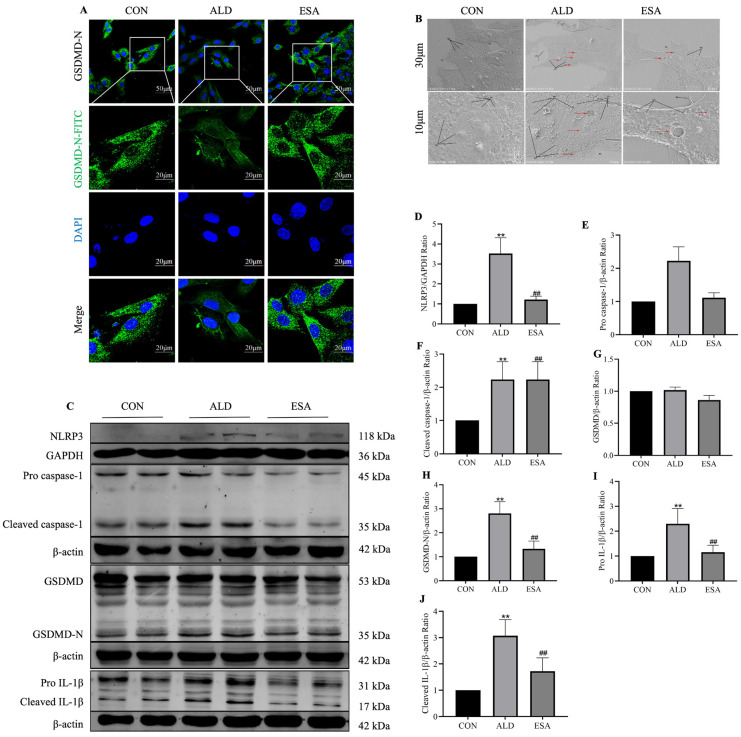
The expression of pyroptosis-related molecules in aldosterone-treated rat vascular smooth muscle cells (VSMCs). (**A**) Aldosterone-induced GSDMD-targeted cell membrane perforation in VSMCs, GSDMD-N is indicated with FITC (green), and nuclei are stained with DAPI (blue). Scale bars = 20 μm and 50 μm. (**B**) Scanning electron microscopy (SEM) results showing the pyroptosis of VSMCs. Scale bars = 30 μm and 10 μm. Mv: microvilli; Ps: pseudopodia; CP: cell protrusions. Red arrows point to pores or pits across the cell surface. (**C**–**J**) The protein expression of NLRP3, pro-caspase-1, cleaved caspase-1, GSDMD, GSDMD-N, pro-IL-1β, and cleaved IL-1β in aldosterone-treated VSMCs was detected using Western blotting. *n* = 6. The values are the means ± SDs, ** *p* < 0.01 compared to CON, and ^##^ *p* < 0.01 compared to ALD. ALD: rat VSMCs were treated with aldosterone (10^−7^ mol/L) for 24 h. ESA: rat VSMCs were pretreated with 10^−6^ mol/L esaxerenone for 2 h prior to aldosterone treatment.

We performed in vitro experiments to further verify whether aldosterone can induce pyroptosis in VSMCs. We examined the effect of aldosterone on VSMC pyroptosis using immunofluorescence staining, SEM, and Western blotting. The immunofluorescence results revealed that the expression of GSDMD-N in the cell membrane increased after stimulation with aldosterone (Figure 3A). Scanning electron microscopy (SEM) revealed that the overall structure of the cells in the CON group was acceptable, the number of microvilli (Mv) on the surface of the cell membrane was abundant, and the cell pseudopodia (Ps) structure was normal. In the ALD group, the cells exhibited changes consistent with pyroptosis; as the cells were moderately edematous, several pores or pits of different sizes were visible across the cell surface (red arrows), many cell protrusions (CPs) formed vesicle structures, and the cell Ps degenerated (Figure 3B). Western blotting revealed that the protein expression of NLRP3, pro-caspase-1, cleaved caspase-1, GSDMD-N, pro-IL-1β, and cleaved IL-1β was increased after aldosterone treatment (*p* < 0.01, Figure 3C–J). GSDMD protein expression in the ALD group was not significantly different from that in the CON group (*p* > 0.05). After esaxerenone treatment, the expression of GSDMD-N in the cell membrane was attenuated, and the protein expression levels of the indicators listed above, except for GSDMD, were decreased (*p* < 0.01). Moreover, the degree of cell swelling and the formation of fenestrae in the cell membrane in the ESA group were lower than those in the ALD group, and the degeneration of cell Ps was significantly alleviated (Figure 3B). Taken together, these results suggest that esaxerenone inhibits aldosterone-induced pyroptosis in VSMCs.

### 3.4. Aldosterone Induces DNA Damage in VSMCs

**Figure 4:**
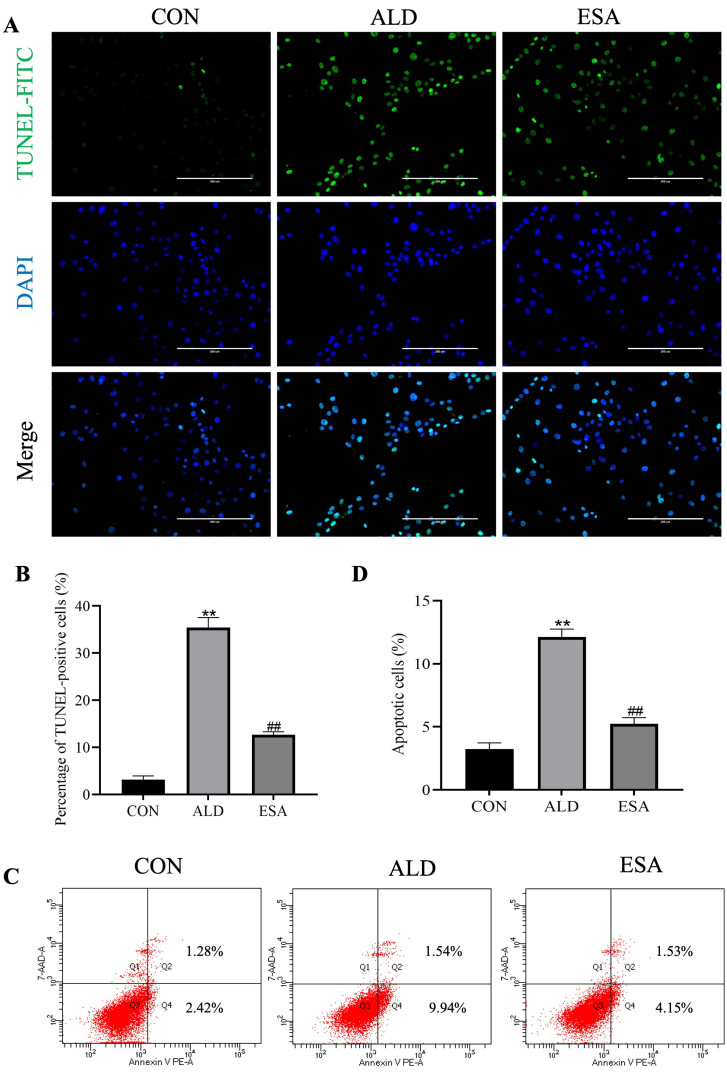
Aldosterone-induced DNA damage in rat vascular smooth muscle cells (VSMCs). (**A**,**B**) TUNEL staining was used to detect aldosterone-induced DNA damage in VSMCs. TUNEL staining is indicated with FITC (green), and nuclei were stained with DAPI (blue). Scale bar = 200 μm. (**C**,**D**) Flow cytometry was used to detect aldosterone-induced DNA damage in VSMCs. *n* = 6. The values are the means ± SDs, ** *p* < 0.01 compared to CON, and ^##^ *p* < 0.01 compared to ALD. ALD: rat VSMCs were treated with aldosterone (10^−7^ mol/L) for 24 h. ESA: rat VSMCs were pretreated with 10^−6^ mol/L esaxerenone for 2 h prior to aldosterone treatment. PE Annexin V: phycoerythrin-conjugated Annexin V. 7-AAD: 7-amino-actinomycin. The cells that were considered viable were Annexin V- and 7-AAD-negative, cells that were in early apoptosis were Annexin V-positive and 7-AAD-negative, and cells that were in late apoptosis or already dead were both Annexin V- and 7-AAD-positive.

We stimulated VSMCs with aldosterone at different time points and used immunofluorescence staining to assess the level of the DNA damage marker γ-H2AX to observe aldosterone-induced changes in DNA damage in VSMCs. Phosphorylated H2AX, or γ-H2AX, is the product formed after DNA double-stranded breaks and can reflect the degree of DNA damage and repair. The results revealed that the DNA began to be significantly damaged after 24 h. Compared to that in the CON group, the DNA damage in the ALD group was more obvious at 6 h, and the degree of DNA damage in the CON group subsequently remained stable, while the number of cells with DNA damage in the ALD group gradually increased, reaching a peak at 24 h and remaining high at 36 h (Figure S1). We examined the degree of DNA damage in VSMCs using TUNEL staining and flow cytometry to further determine the occurrence of pyroptosis. Compared to the percentage of TUNEL-positive cells in the CON group (3.164 ± 0.791%), the percentage in the ALD group (35.431 ± 2.108%) was significantly greater (*p* < 0.01, Figure 4A,B). Furthermore, the flow cytometry results confirmed a greater percentage of apoptotic cells in the ALD group (CON group vs. ALD group, 3.247 ± 0.486% vs. 12.123 ± 0.632%, *p* < 0.01; Figure 4C,D). Notably, esaxerenone inhibited aldosterone-induced DNA damage (ALD group vs. ESA group, 35.431 ± 2.108% vs. 12.698 ± 0.605%, *p* < 0.01) and decreased the percentage of apoptotic cells (ALD group vs. ESA group, 12.123 ± 0.632% vs. 5.228 ± 0.5%, *p* < 0.01; Figure 4). Taken together, these results suggest that aldosterone causes not only VSMC pyroptosis but also DNA damage.

### 3.5. Mitochondrial Damage in Aldosterone-Induced VSMCs

**Figure 5:**
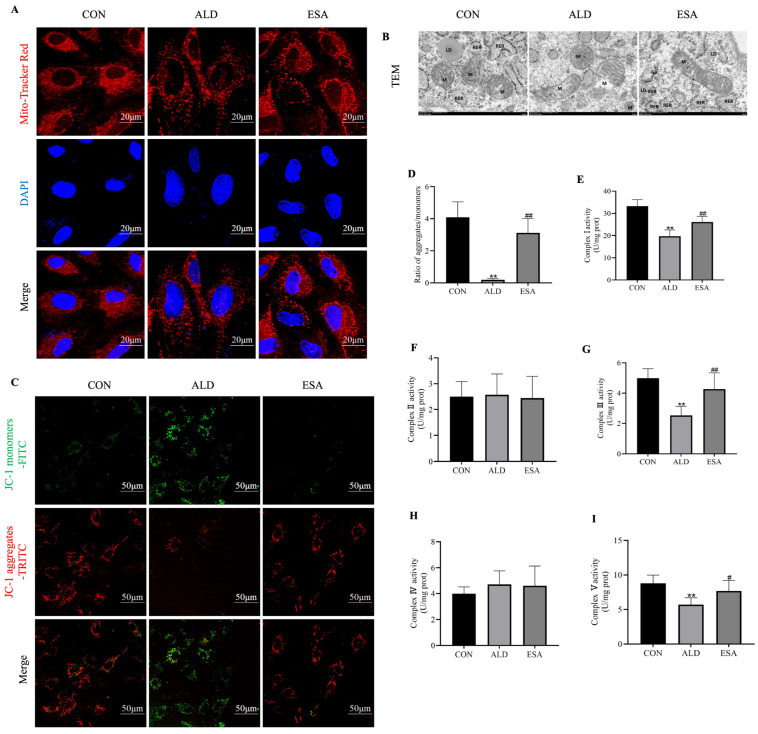
Mitochondrial damage in aldosterone-treated rat vascular smooth muscle cells (VSMCs). (**A**) Immunofluorescence staining of aldosterone-induced mitochondrial changes in VSMCs. The MitoTracker was labeled with TRITC (red), and the nuclei were stained with DAPI (blue). (**B**) SEM results showing aldosterone-induced changes in the mitochondrial ultrastructure of VSMCs. M: mitochondria. RER: rough endoplasmic reticulum. LD: lipid droplets. Go: Golgi apparatus. Scale bars = 20 μm (immunofluorescence staining) and 500 nm (TEM). (**C**,**D**) Changes in the mitochondrial membrane potential of VSMCs, JC-1 monomers (FITC, green), and JC-1 aggregates (TRITC, red) are shown. Scale bar = 50 μm. (**E**–**I**) Changes in mitochondrial respiratory chain complex (COX) I-V activity in VSMCs. The values are the means ± SDs, ** *p* < 0.01 compared to CON, ^#^ *p* < 0.05 compared to ALD, and ^##^ *p* < 0.01 compared to ALD. ALD: rat VSMCs were treated with aldosterone (10^−7^ mol/L) for 20 min (for the detection of ROS) or 24 h. ESA: rat VSMCs were pretreated with 10^−6^ mol/L esaxerenone for 2 h prior to aldosterone treatment.

We examined VSMCs to determine whether aldosterone-induced VSMC damage is related to mitochondria. The MitoTracker Red CMXRos results revealed that, after aldosterone stimulation, the mitochondria appeared damaged, changing from normal long rods to puncta (Figure 5A). Next, we observed the ultrastructure of VSMC mitochondria using TEM, and the results revealed that the mitochondria were severely swollen after aldosterone stimulation of VSMCs in vitro. The intramembrane matrix was dissolved and ultimately disappeared, and the cristae were broken and/or missing (Figure 5B). We used a JC-I probe to detect changes in the cell membrane potential and assess mitochondrial function and found that the mitochondrial membrane potential decreased after aldosterone stimulation (Figure 5C,D). Changes in COX I–V activity were assessed, and the results revealed that the activities of COX I (CON group vs. ALD group, 33.236 ± 2.950 vs. 19.695 ± 2.869, *p* < 0.01), COX III (CON group vs. ALD group, 4.993 ± 0.614 vs. 2.526 ± 0.598, *p* < 0.01), and COX V (CON group vs. ALD group, 8.783 ± 1.213 vs. 5.714 ± 0.975, *p* < 0.01; Figure 5E–I) decreased after the addition of aldosterone. However, the activities of COX II and COX IV did not change significantly (*p* > 0.05). These data suggest that aldosterone-induced mitochondrial damage may occur through COX I, III, and V. After treatment with esaxerenone, the mitochondria recovered to a long rod shape and were slightly swollen. Some mitochondrial membrane structures were partially dissolved and blurred, and the cristae were neatly arranged. Furthermore, esaxerenone reversed the decrease in the membrane potential and inhibited the decreases in the activities of COX I (ALD group vs. ESA group, 19.695 ± 2.869 vs. 26.162 ± 2.530, *p* < 0.01), COX III (ALD group vs. ESA group, 2.526 ± 0.598 vs. 4.272 ± 1.065, *p* < 0.01), and COX V (ALD group vs. ESA group, 5.714 ± 0.975 vs. 7.685 ± 1.501, *p* < 0.05).

**Figure 6:**
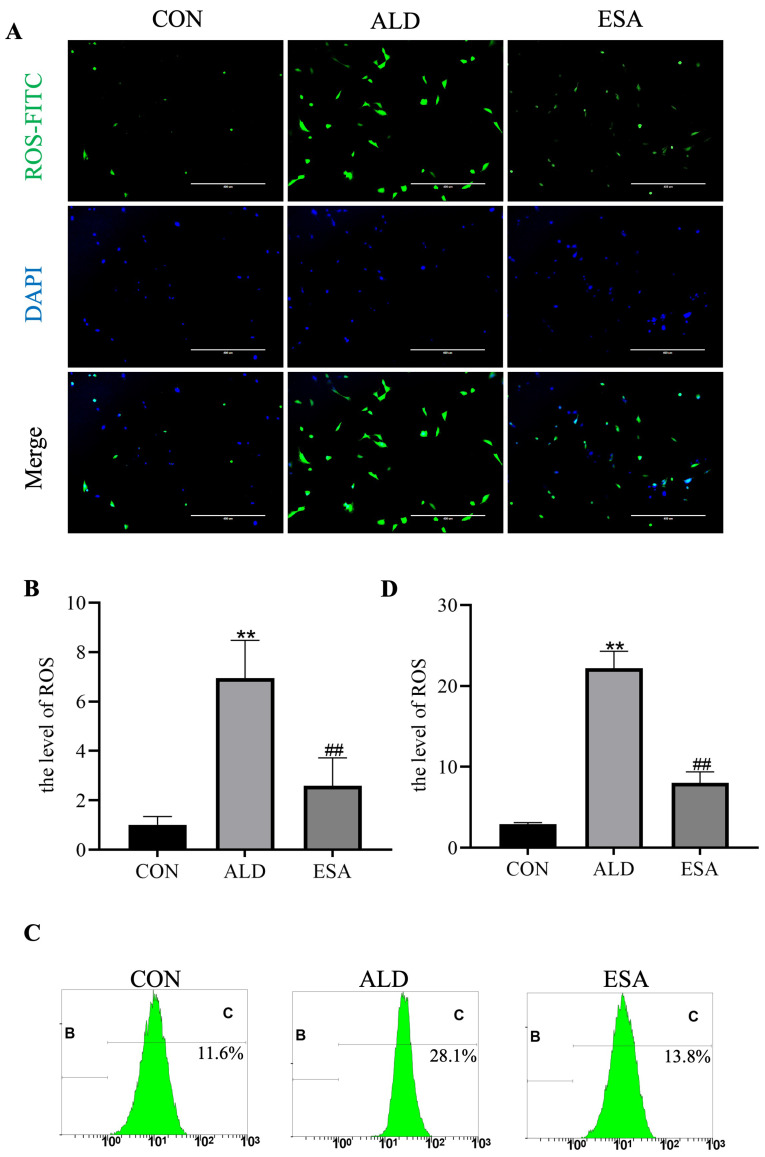
Changes in ROS levels in aldosterone-treated rat vascular smooth muscle cells (VSMCs). (**A**,**B**) Changes in intracellular ROS levels in VSMCs detected using immunofluorescence staining. The ROS were labeled with FITC (green), and nuclei were stained with DAPI (blue). Scale bar = 400 μm (immunofluorescence staining). *n* = 6. (**C**,**D**) Flow cytometric detection of ROS levels in VSMCs. B: undetectable fluorescence areas (negative areas). C: detectable fluorescence area (positive area). *n* = 3. The values are the means ± SDs, ** *p* < 0.01 compared to CON, and ^##^ *p* < 0.01 compared to ALD. ALD: rat VSMCs were treated with aldosterone (10^−7^ mol/L) for 20 min (for the detection of ROS) or 24 h. ESA: rat VSMCs were pretreated with 10^−6^ mol/L esaxerenone for 2 h prior to aldosterone treatment.

The mitochondria are the main site of ROS production, which are important for oxidative damage; therefore, we detected changes in the intracellular ROS concentration. The immunofluorescence results revealed that the ROS content was 1.014 ± 0.333% in the CON group, 6.948 ± 1.532% in the ALD group, and 2.593 ± 1.127% in the ESA group (Figure 6A,B). Flow cytometry further validated this result (Figure 6C,D). Overall, aldosterone causes mitochondrial damage and subsequently increases ROS levels, which may be an important mechanism of aldosterone-induced VSMC damage.

### 3.6. Aldosterone Induces Mitochondrial Damage and Leads to VSMC Pyroptosis by Activating MRs

**Figure 7:**
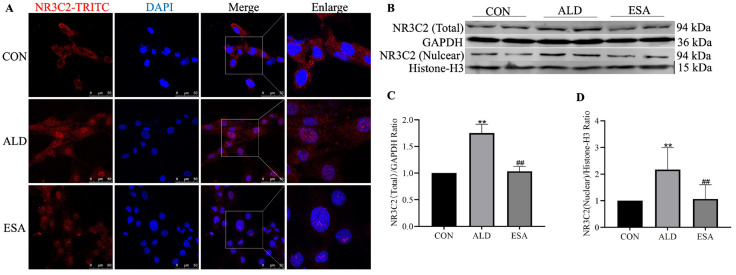
Effects of esaxerenone on MRs (encoded by NR3C2) in aldosterone-treated rat vascular smooth muscle cells (VSMCs). (**A**) Immunofluorescence staining of VSMCs with antibodies against NR3C2 (TRITC, red). Nuclei were stained with DAPI (blue) to visualize the nuclear translocation of NR3C2 in rat VSMCs. Scale bar = 50 μm. (**B**–**D**) Esaxerenone inhibited aldosterone-induced upregulation of total (*p* < 0.01) and nuclear (*p* < 0.01) NR3C2 protein expression in rat VSMCs, as detected using Western blotting. *n* = 6. The values are the means ± SDs, ** *p* < 0.01 compared to CON, and ^##^ *p* < 0.01 compared to ALD. ALD: rat VSMCs were treated with aldosterone (10^−7^ mol/L) for 24 h. ESA: rat VSMCs were pretreated with 10^−6^ mol/L esaxerenone for 2 h prior to aldosterone treatment.

The mechanism of aldosterone-induced mitochondrial damage was further verified by detecting the nuclear translocation and expression of the MR protein (encoded by NR3C2). The immunofluorescence results revealed that the expression of NR3C2 in the nucleus was significantly increased after aldosterone stimulation (Figure 7A). Western blotting revealed that, compared to the CON group, the total and nuclear NR3C2 protein levels were increased in the ALD group (*p* < 0.01, Figure 7B–D). However, esaxerenone treatment inhibited the nuclear translocation and expression of the NR3C2 protein (*p* < 0.01, Figure 7). These results suggest that aldosterone induces mitochondrial damage through MR activation and nuclear translocation, thereby causing VSMC pyroptosis.

## 4. Discussion

This study provides strong evidence for the involvement of aldosterone in the pathogenesis of aortic injury by activating MR-induced pyroptosis in VSMCs and provides insights into the role of esaxerenone in protecting the aorta by inhibiting mitochondrial oxidative damage and VSMC pyroptosis. These conclusions are based on the following findings: (1) aortic damage and VSMC pyroptosis were observed in an aldosterone-infused mouse model and in cultured VSMCs stimulated with aldosterone, (2) mitochondrial oxidative damage was involved in VSMC pyroptosis, and (3) esaxerenone attenuated mitochondrial and DNA damage by inhibiting MR activation, thereby inhibiting aldosterone-induced pyroptosis in VSMCs and protecting the aorta.

The mechanisms of vascular disease are complex and include immune cell infiltration, vascular aging, calcification, atherosclerosis, and extracellular matrix remodeling [23]. Regardless of etiology, the occurrence of CVD is strongly associated with inflammation, which involves immune cells, platelets, lipid oxidation, and smooth muscle cells [8,24]. In addition, risk factors for the development of cardiovascular disease may reduce the detoxification capacity of the antioxidant defense system and stimulate excessive reactive oxygen species (ROS) and reactive nitrogen species (RNS) production. Oxidative stress leads to damage to lipids, proteins, DNA molecules, and cell membranes while also reducing the availability of nitric oxide (NO) and triggering early events in the pathogenesis of CVD and many other chronic degenerative diseases [24,25]. Aldosterone, on the other hand, is involved in inflammation in various tissues, such as the kidney and heart [21,26,27,28]. Increasing evidence suggests that elevated aldosterone levels and consequent MR overactivation may be involved in the pathogenesis and progression of a variety of cardiovascular diseases, including hypertension and coronary artery disease [15,16]. Excessive aldosterone secretion can lead to vascular damage, dysfunction, and remodeling [29]. We used a 12-week aldosterone-treated mouse model in this study to validate the effect of aldosterone on the vascular inflammatory response. One unexpected finding was that aldosterone perfusion alone did not induce hypertension, which is also similar to the findings of a previous study in C57B6 mice [30] and may be related to the absence of kidney resection, but the results showed that elevated aldosterone levels did induce changes in aortic damage. In addition, we found that VSMCs in the aorta were also damaged, and TUNEL staining and TEM also revealed DNA and mitochondrial damage in the VSMCs (Figure 1).

VSMCs constitute a large percentage of the medial layer of the vascular wall and not only play an important role in maintaining vascular homeostasis but also contribute to vascular diseases in various ways [31,32]. Pyroptosis is a proinflammatory form of cell death that is associated with the pathogenesis of cardiovascular diseases, including atherosclerosis, hypertension, myocardial infarction, and diabetic cardiomyopathy [8,31,33]. Pyroptosis has been investigated in relation to CVD, and mainly involves monocytes, macrophages, vascular endothelial cells, cardiac fibroblasts, cardiomyocytes, and other cell types [8]. VSMC pyroptosis has been reported to contribute to the pathogenesis of atherosclerosis [34]. The interaction between VSMC death and inflammation is also a key factor in the progression of vascular calcification [35]. These findings suggest that aldosterone may induce VSMC pyroptosis and cause further aortic damage.

We performed in vivo and in vitro experiments to verify the role of aldosterone in VSMC pyroptosis. Caspase-1-mediated cleavage of GSDMD is a necessary step for triggering pyroptosis; thus, we examined the expression of related proteins in aldosterone-treated mouse aortas and aldosterone-stimulated VSMCs. As shown in Figure 2, both the immunohistochemistry and Western blotting results revealed that the expression of the pyroptosis-related proteins NLRP3, cleaved caspase-1, GSDMD-N, and IL-1β was increased in the ALD group, and these changes indicate the occurrence of aortic VSMC pyroptosis, suggesting that aortic injury due to VSMC pyroptosis may be related to aldosterone levels. We treated cells with aldosterone to further clarify the role of aldosterone in VSMC pyroptosis, and the immunofluorescence and SEM results revealed that GSDMD-N was transferred to the cell membrane and formed pores. The Western blotting results also confirmed the increased expression of pyroptosis-related proteins (Figure 3). Moreover, TUNEL staining and flow cytometry revealed that the percentage of TUNEL-positive cells and the percentage of apoptotic cells were significantly increased after aldosterone stimulation, which confirmed that aldosterone induces cell death by causing cellular DNA damage and increasing the rate of apoptosis through MR activation (Figure 4). These results are consistent with the SEM and histochemical staining results, confirming that aldosterone can induce pyroptosis in VSMCs.

We next investigated the underlying mechanism by which aldosterone can cause aortic damage by inducing VSMC pyroptosis. Previous studies have shown that NLRP3 inflammasome activation may be a major trigger of vascular injury associated with excess aldosterone [2,29]. Many potential factors underlie NLRP3 inflammasome activation, such as K^+^ efflux, endoplasmic reticulum stress, NADPH oxidase, and mitochondrial dysfunction (MtD) [36]. Numerous studies have confirmed that, in endothelial cells, cardiomyocytes, and VSMCs, various stimuli lead to MtD and ROS overproduction, which, in turn, induces NLRP3 inflammasome activation and pyroptosis [8]. MR can also cause MtD in a diabetic state and high-glucose conditions via the PI3K/Akt/eNOS signaling pathway [37]. MtD manifests as the overproduction of ROS in mitochondria, mitochondrial DNA (mtDNA) damage, and respiratory chain dysfunction [38]. Oxidized mtDNA generation and the massive accumulation of ROS can trigger the activation of the NLRP3 inflammasome and subsequently induce caspase-1-dependent pyroptosis [33,39]. The results of our study are also consistent with the fact that aldosterone activates MRs, induces mitochondrial damage and ROS overproduction, activates the NLRP3 inflammasome, and induces pyroptosis in VSMCs, leading to vascular damage. After aldosterone stimulation, the morphology of the mitochondria was altered (Figure 4), and mitochondrial function was impaired (Figure 5). Mitochondrial dysfunction subsequently induces ROS production [8], which was also confirmed in our study through immunofluorescence staining and flow cytometry (Figure 6). Other studies have confirmed that GSDMD is part of the de novo mechanism by which mitochondrial ROS promote NLRP3 inflammasome-dependent pyroptotic cell death [39]. In addition, GSDMD-induced pyroptosis leads to mitochondrial damage, N-terminal pore-forming GSDMD fragments (GSDMD-NT) rapidly destroy mitochondria, and mitochondrial damage plays a crucial role in pyroptosis [40]. Whether GSDMD causes mitochondrial damage in aldosterone-treated mice and subsequently participates in VSMC pyroptosis requires further verification in the future.

Aldosterone binds to and activates MRs, and the aldosterone–MR complex is translocated to the nucleus [41]. The present study confirmed that aldosterone can upregulate the level of NR3C2, stimulate the nuclear translocation (Figure 7), and induce VSMC pyroptosis; therefore, blocking MR activation and inhibiting VSMC pyroptosis are potential treatments for reducing cardiovascular complications. Previous studies have shown that MR blockers affect several extrarenal tissues, including vascular tissues [16]. Spironolactone and eplerenone are traditionally available MR blockers, but their clinical use has been limited because of their effects on estrogen and glucocorticoid receptors. Compared to these two agents, esaxerenone has shown good antihypertensive activity in a variety of patients, with strong antihypertensive and renoprotective effects, and its suppressive effect is more potent and longer lasting [42]. In addition, esaxerenone has anti-inflammatory effects [19]. However, the effect of esaxerenone on vascular function has not been fully investigated. This study focused on the role of esaxerenone in aldosterone-induced aortic VSMC pyroptosis. The experimental results showed that esaxerenone inhibited MR activation and nuclear translocation, alleviated mitochondrial damage and ROS accumulation, and reduced VSMC pyroptosis, thereby exerting its role in protecting blood vessels. These results also confirm the findings of previous studies in which aldosterone and MR negatively affected the survival, growth, and function of cardiomyocytes and vascular cells (VSMCs and endothelial cells) and induced adverse structural remodeling of the vasculature, with actions that were almost abolished by MR blockers [16,43]. These findings also indicate the important role of esaxerenone in aldosterone-induced VSMC pyroptosis and support our findings from aldosterone-perfused mice and cultured VSMCs.

This study has several limitations. Hyperaldosteronism not only causes hypertension and cardiovascular disease but also causes electrolyte disturbances [44], and upstream signals of the NLRP3 inflammasome also include the efflux of potassium ions (K^+^) or chloride ions (Cl^−^) and the flux of calcium ions (Ca^2+^) [45]. Our previous study revealed that the serum potassium levels were reduced in aldosterone-perfused mice [21]. However, whether electrolyte changes affect or participate in aldosterone-induced VSMC pyroptosis requires further study. Due to limited experimental conditions, we used mice as a model for in vivo experiments and rat VSMCs for in vitro tests. The use of allogeneic animals and cells may increase the robustness of our observations. In addition, whether the mechanism of action of esaxerenone involves immune cells and the detailed mechanism of action of esaxerenone in mitochondrial damage also need to be further verified.

## 5. Conclusions

In conclusion, we performed in vivo experiments on mice and in vitro experiments in rat VSMCs and showed that aldosterone induces mitochondrial oxidative damage and pyroptosis in VSMCs and participates in aortic injury and that the MR blocker esaxerenone antagonizes these effects. These findings suggest that targeting the aldosterone/MR pathway may be an effective therapeutic strategy for aortic injury and hypertension. Additionally, esaxerenone, a novel nonsteroidal MR blocker, has high research value in this regard.

## Data Availability

The data from the current study are available from the corresponding author upon reasonable request.

## Supplementary Materials

The following supporting information can be downloaded at: https://www.mdpi.com/article/10.3390/life14080967/s1. Figure S1. Changes in DNA damage caused by aldosterone stimulation of vascular smooth muscle cells (VSMCs) at different times.

## Abbreviations

VSMC: vascular smooth muscle cell; TEM: transmission electron microscopy; SEM: scanning electron microscopy; CVD: cardiovascular disease; mtROS: mitochondrial ROS; NLRP3: NOD-like receptor thermal protein domain-associated protein 3; IL-1β: interleukin-1 beta; MR: mineralocorticoid receptor; CON: control; ALD: aldosterone; ESA: esaxerenone; FBS: fetal bovine serum; DMSO: dimethyl sulfoxide; PFA: paraformaldehyde; H&E: hematoxylin and eosin; NR3C2: nuclear receptor subfamily 3 group C member 2; caspase: cysteinyl aspartate specific proteinase; GSDMD: gasdermin D; PB: phosphate buffer; PBS: phosphate-buffered saline; PE: phycoerythrin; ROS: reactive oxygen species; RIPA: radioimmunoprecipitation assay; PVDF: polyvinylidene fluoride; GAPDH: glyceraldehyde-3-phosphate dehydrogenase; Mv: microvilli; Ps: pseudopodia; CP: cell protrusions; MtD: mitochondrial dysfunction; mtDNA: mitochondrial DNA; SD: standard deviation; ANOVA: analysis of variance.
