# Supplementary material for: Esaxerenone Attenuates Aldosterone-Induced Mitochondrial Damage-Mediated Pyroptosis in Mouse Aorta and Rat Vascular Smooth Muscle Cells
Source: Life (Basel). 2024 Jul 31;14(8):967. doi: 10.3390/life14080967 (PMC11355590; doi:10.3390/life14080967)
Supplement: Supplementary file 1 [file life-14-00967-s001.zip › life-3108352-supplementary.docx]

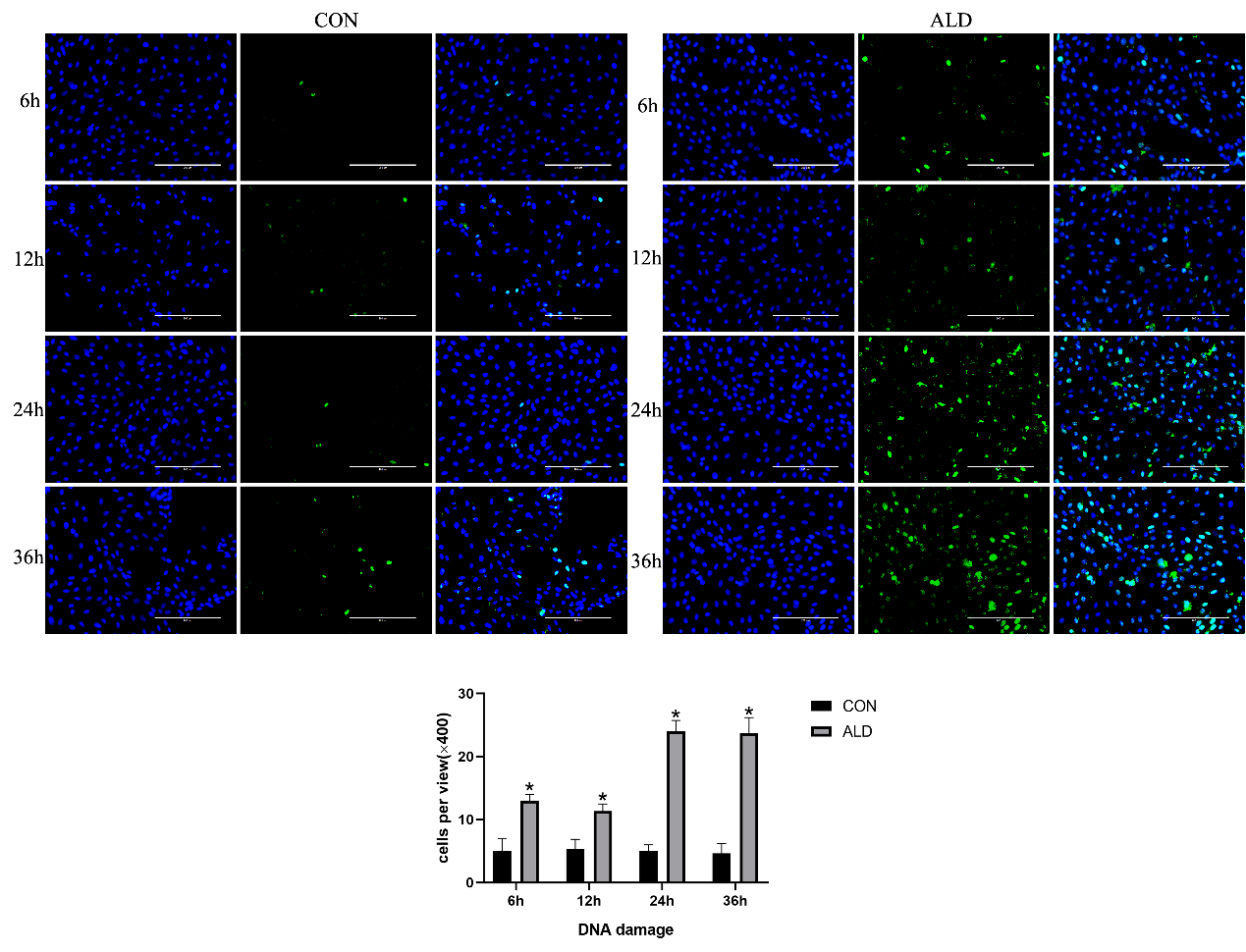


**Supplement Figure S1 Changes in DNA damage caused by aldosterone stimulation of vascular smooth muscle cells (VSMCs) at different times.**

Changes in γ-H2AX expression of VSMCs at different time points. γ-H2AX stained with green fluorescence, DAPI staining of nuclei is blue fluorescence. Scale bar = 200 μm. *n* = 3. Values are the mean ± SD, ^*^*P* < 0.05 compared to CON. ALD: rat VSMCs were treated with aldosterone (10^-7^ mol/L) for 6, 12, 24, 36 hours.
